# Universal quantum gates for hybrid system assisted by atomic ensembles embedded in double-sided optical cavities

**DOI:** 10.1038/srep43675

**Published:** 2017-03-08

**Authors:** A.-Peng Liu, Liu-Yong Cheng, Qi Guo, Shou Zhang, Ming-Xia Zhao

**Affiliations:** 1 Shanxi Institute of Technology, Yangquan, Shanxi 045000, China; 2 School of Physics and Information Engineering, Shanxi Normal University, Linfen, Shanxi 041004, China; 3 College of Physics and Electronics Engineering, Shanxi University, Taiyuan, Shanxi 030006, China; 4 Department of Physics, College of Science, Yanbian University, Yanji, Jilin 133002, China

## Abstract

We propose deterministic schemes for controlled-NOT (CNOT), Toffoli, and Fredkin gates between flying photon qubits and the collective spin wave (magnon) of an atomic ensemble inside double-sided optical microcavities. All the gates can be accomplished with 100% success probability in principle and no additional qubit is required. Atomic ensemble is employed so that light-matter coupling is remarkably improved by collective enhancement. We qualified the performance of the gates and the results show that they can be faithfully constituted with current experimental techniques.

Quantum logic gates usually lie at the heart of quantum-information processing (QIP) tasks. As is well known, any *n*-qubit quantum operation can be decomposed into combinations of two-qubit gates and single-qubit operations^1^. So far, it has been well solved for the optimal synthesis of two-qubit gates, while it is more complex and still an open question for the case of multi-qubit systems. So it is of significance to find a simpler way for directly implementing multi-qubit gates. On the other hand, Toffoli and Fredkin gates are fundamental quantum gate for three-qubit systems, and they have attracted much attention since they can form a universal quantum computation architecture together with single-qubit operations^2^,^3^,^4^,^5^,^6^,^7^. Moreover, they play an important role in quantum algorithms^8^, entanglement concentration and purification^9^,^10^,^11^, error correction^12^, and fault-tolerant quantum circuits^13^. Many proposals have been proposed to implement quantum logic gates with several physical systems theoretically and experimentally, such as the ion trap^14^, nuclear magnetic resonance^15^,^16^, quantum dot (QD)^17^,^18^,^19^, superconducting qubits^20^,^21^, nitrogen-vacancy (NV) centers^22^,^23^, and photon systems^24^,^25^.

For scalable quantum computation and QIP, quantum gates between two separated quantum nodes are indispensable. So far, one convenient way to realize such gates is to use linked cavities, each of which contains single or several qubits in it. To constitute the critical two-qubit optical gate in a deterministic way, one can resort to Kerr nonlinearities. However, they are many orders of magnitude too small for efficient quantum computation for naturally occurring nonlinearities in the single-photon level^26^. Several proposals based on Kerr nonlinearities in fibers or crystals^27^, electromagnetically induced transparency^22^,^28^,^29^,^30^, and optical dipole-cavity system^31^,^32^ are developed. In the past decades, cavity quantum electrodynamics (cavity QED) that studies the coherent interaction of matter with quantized fields has been a paradigm for QIP due to controllable interactions between dipole and photons^31^,^33^. As for the cavity-based scheme, the dipole embedded in the optical cavity interacts strongly with the input single photons, and the interaction between the dipole and the successive photons provides strong Kerr nonlinearities^17^,^18^,^31^,^34^.

In 2004, Duan *et al*.^31^ proposed a scheme for scalable photonic quantum computation based on cavity-assisted interaction between single-photon pulses. In 2005, Cho *et al*.^32^ proposed a scheme to implement a two-qubit controlled-phase gate for single atomic qubits based on the cavity input-output process. Based on a singly charged QD inside an optical resonant cavity, several schemes for entanglement generation and implementing of quantum logic gates are proposed^17^,^18^,^19^. Assisted with single photons, Zhou *et al*.^35^ provided the optimal approach to detect nonlocal atomic entanglement. On the other hand, based on the photonic Faraday rotation, they also described the complete logic Bell-state analysis^36^. With the dipole induced transparency of a diamond NV center, universal hyperparallel hybrid photonic quantum logic gates were proposed in 2015^22^. Recently, an magnon-cavity unit, e.g., an atomic ensemble confined in a double-sided cavity, was proposed by Li *et al*.^34^, in which the interaction between the collective spin wave (magnon) of an atomic ensemble and the successive photons provides strong Kerr nonlinearities.

In this paper, inspired by the above works, we investigate the possibility of achieving scalable photonic quantum computation assisted by an atomic ensemble in a double-sided cavity. Our schemes are different from the work by Li *et al*.^34^ in which they present a scheme for two CNOT gates with the photonic qubits both in the spatial degrees of freedom (DOF) and the polarization DOF of each photon. By the nonlinear interaction between the moving photon and the magnon of an atomic ensemble in a double-sided cavity, we first present a deterministic scheme for constructing a CNOT gate on a hybrid system with the flying photon as the control qubit and the atomic ensemble as the target qubit. Besides, we construct the Toffoli and Fredkin gates on a three-qubit hybrid system in a deterministic way. In our work, the control qubit of our universal gates is encoded on the polarization states of the moving photon, while the target qubit is encoded on the state of atomic ensemble inside an optical microcavity. These three schemes for the universal gates require no additional qubit, and they only need some linear optical elements besides Kerr nonlinear interaction between the magnon and the photons. High fidelities and high efficiencies can be achieved in the strong coupling regime and are not sensitive to the frequency detuning and coupling imbalance.

## Results

### Input-output relation for a single photon with a magnon-cavity coupling system

**Figure 1:**
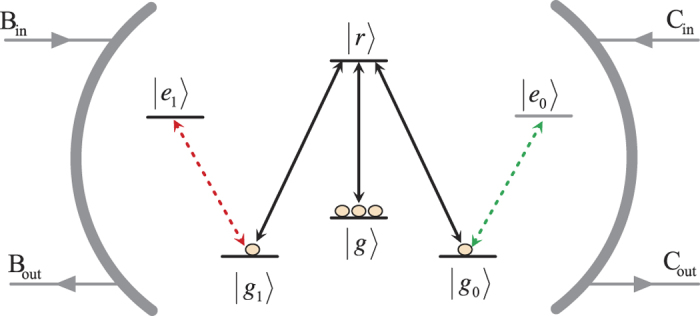
Schematic diagram of the atomic ensemble cavity coupling system.

The configuration of the atomic ensemble cavity coupling system considered here is exhibited schematically in Fig. 1. We first denote a highly excited Rydberg state as |*r*〉. Assisted by the Rydberg state |*r*〉, one can prepare the atomic ensemble into the magnon state and perform the single-qubit operation on the magnon qubit. A qubit is encoded in collective spin wave state or magnon state with a single atom in the states |*g*_0_〉 and |*g*_1_〉 of the atomic ensemble. If we define 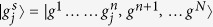 (*j* = 0, 1), we have 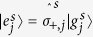, where 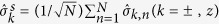 are the collective angular momentum operators with 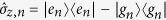, 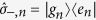 and 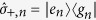. The transitions 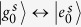 and 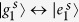 with frequency *ω*_0_ are driven by orthogonal polarizations (*H* and *V*) of a photon with frequency *ω*. Meanwhile the two transitions are nearly resonantly coupled to the two degenerate cavity modes 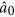 and 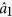 with the corresponding coupling rates are *λ*_0_ and *λ*_1_, respectively. For the input photons with different polarizations, the transmission and reflection coefficients are determined by the state of the ensemble. If a polarized photon is injected into the cavity via either side of the cavity, it will pass through the cavity if it is decoupled from the driven cavity mode; otherwise it will interact with the atomic ensemble if it is coupled to the cavity mode and lead to the mode splitting. When the frequencies of the optical fields close to the cavity frequency *ω*_*a*_, we can take the coupling rates between an asymmetrical cavity and modes 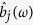 and 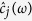 of ports *B* and *C* as real constant^31^. Here, to insure the photon pulse shape remains unchanged, we need a single polarized photon pulse with a finite bandwidth ([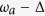, 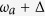]), which is satisfied when 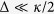 (the cavity decay rate)^17^,^18^. If we take *ω*_*a*_ as the carrier frequency, then *δ*′ = *ω* − *ω*_*a*_ denotes the frequency detuning of the input photon with frequency *ω. δ*_0_ = *ω*_0_ − *ω*_*a*_ measures the frequency difference between the dipole transition and the cavity mode. This system exhibits similar features with the Jaynes-Cummings model, and in the frame rotating with respect to *ω*_*a*_, the dynamics of the system is governed by the following hamiltonian (*ħ* = 1)^31^,^33^,^37^


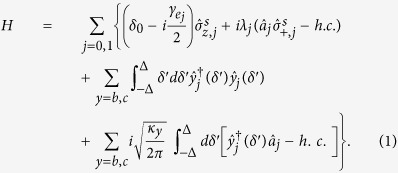


here 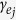 and *λ*_*j*_ denote the spontaneous emission rate of the single excited collective state 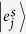 and the coupling rate between the atomic ensemble and the corresponding resonant cavity mode, respectively. With the help of Rydberg state^38^,^39^ or coherent Raman process^40^,^41^, one can pump the atomic ensemble to the magnon state 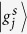, so that the input photon will drive the interaction between the atomic ensemble and the cavity mode. In the single excitation subspace, the system will evolve in the space spanned by the internal states of the atomic ensemble and the photon number states of the radiation modes (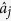, 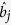, and 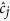), respectively. Suppose the initial state of the system is 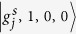, i.e., we choose the input photon in mode 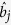, then the state of the system, at time *t*, will evolve to


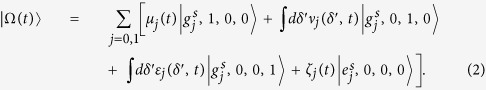


The Schrödinger equation for this system can be specified to be


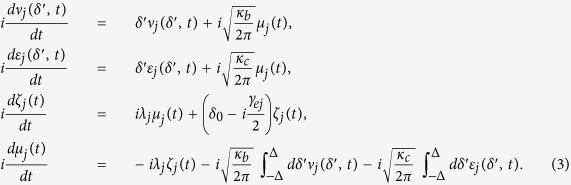


Along with the standard input-output relation 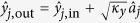 (*y* = *b, c*), we can see the birefringent character of the magnon-cavity system. Here 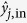 and 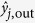 are the input and output field operators, respectively. Under the condition that the incoming field is very weak, i.e., we take 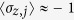, the reflection and transmission coefficients of the system can be expressed as


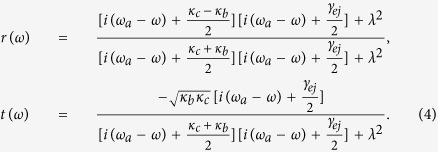


In the case the input photons uncoupled to the cavity, i.e., *λ*_*j*_ = 0, we get the reflection and transmission coefficients for the system, then Eq. (4) reduces to


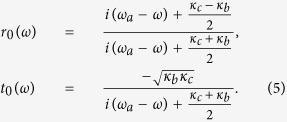


As the backscattering is low in the optical fibers, the asymmetry of the two coupling constants is mainly caused by cavity intrinsic loss^42^. Suppose 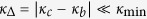 (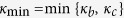), i.e., the difference of the coupling rates between the cavity and the modes 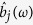 and 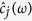 are small, one can replace the reflection and transmission coefficients above for the asymmetrical cavity system with those for the symmetrical one with identical coupling rates, i.e., we set *κ* = *κ*_*b*_ = *κ*_*c*_. With the symmetrical cavity, the corresponding reflection and transmission coefficients can be respectively simplified and given by


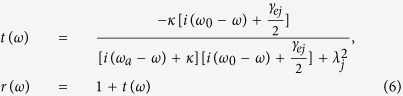


for *λ* > 0 (hot cavity), and


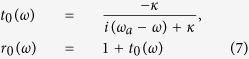


for *λ* = 0 (cold cavity, described with the subscript 0). The reflection and transmission coefficients in Eqs (6) and (7) indicate that the output photon experiences a phase shift relying on the different states of the atomic ensemble in the double-sided cavity. When the Purcell factor *λ*^2^/*κγ* = 1/2, the reflection and transmission coefficients are *r*(*ω*) → 1 and *t*(*ω*) → 0. However, in the decoupling case (*λ* = 0), the reflection and transmission coefficients of the bare cavity are *r*_0_(*ω*) → 0 and *t*_0_(*ω*) → −1. Specifically, if the atomic ensemble is in the state 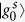 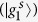, when the photon in |*H*〉 (|*V*〉) state is directed into the cavity, it will be reflected and get no phase shift. Otherwise, the photon will transmit the cavity and get a *π* phase shift. This exactly demonstrates the effective Kerr nonlinearity which can be used to constitute the hybrid multi-qubit gates in the following sections.

### CNOT gate on a two-qubit hybrid system

**Figure 2:**
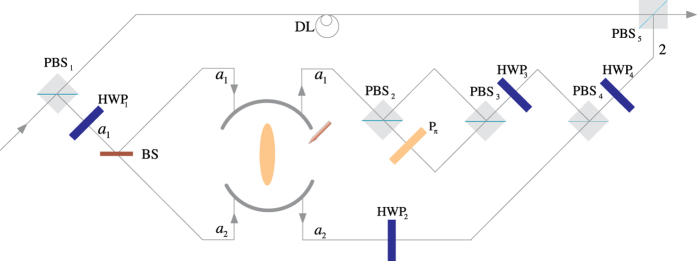
The quantum circuit for constructing a deterministic CNOT gate with a flying photon polarization as the control qubit and a collective spin wave (magnon) qubit as the target qubit. PBS: polarized beam splitter, HWP: half wave plate, BS: beam splitter, *P*_*π*_: phase shifter, DL: delay line.

The framework of our CNOT gate, which flips the target atomic ensemble qubit if the control photon polarization qubit is in the state |*V*〉, is depicted in Fig. 2. The flying photon *p* and the atomic ensemble are prepared in arbitrary superposition states 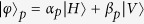 and 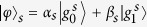 (here 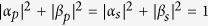), respectively.

For conciseness, we define single-qubit Hadamard operations H_*p*_ and H_*s*_ for one photon and one magnon qubit respectively as:


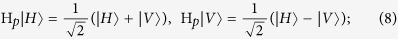



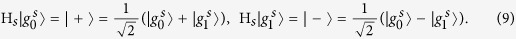


First, the injected photon passes through a polarized beam splitter (PBS_1_), which transmits the photon in the polarization state |*H*〉 and reflects the photon in the state |*V*〉. The part in the state |*H*〉 transmits PBS_1_ and gets into a delay line (DL), does not interact with the cavity, while the part in the state |*V*〉 passes a half-wave plate (HWP_1_), which is used to perform a Hadamard operation (H_*p*_) on the photon. Then the photon passes a beam splitter (BS) and be injected into the cavity from either path *a*_1_ or *a*_2_. At the same time, we perform a Hadamard operation (H_*s*_) on the atomic ensemble with the coherent Raman process or Rydberg-state-assisted quantum rotation. Then the state of the whole system composed of a photon and an atomic ensemble is changed from 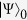 to 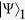. Here


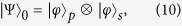


and


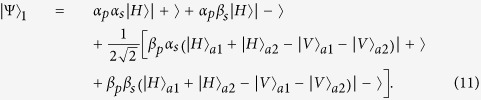


Considering the birefringent propagation of the input polarized photon, the output state of photon together with that of the atomic ensemble is


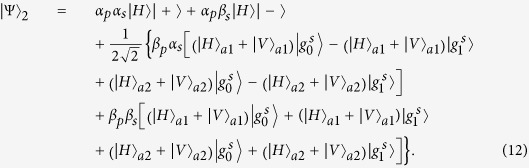


When the photon *p* passes through path *a*_1_, it will be split by PBS_2_, the *H*-polarized component takes a phase shift *π* (i.e., |*H*〉 → −|*H*〉) after passing through the phase shifter *P*_*π*_. Then the photon passes PBS_3_ will take an H_*p*_ operation by HWP_3_. Meanwhile the photon passes through path *a*_2_ will take an H_*p*_ operation by HWP_2_. After the photon passes through PBS_4_ and HWP_4_, the state of the system becomes


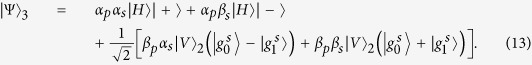


Then we apply an H_*s*_ operation on the atomic ensemble, the state of the hybrid system becomes


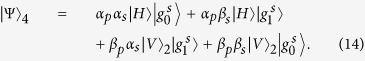


One can see that the state of the atomic ensemble is flipped when the photon (the control qubit) is in the state |*V*〉, while it does not change when the photon is in the state |*H*〉, compared to the original state of the two-qubit hybrid system shown in Eq. (10). Therefore, the quantum circuit shown in Fig. 2 can be used to construct a deterministic CNOT gate with a success probability of 100% in principle by using the photon as the control qubit and the atomic ensemble as the target qubit.

### Toffoli gate on a three-qubit hybrid system

**Figure 3:**
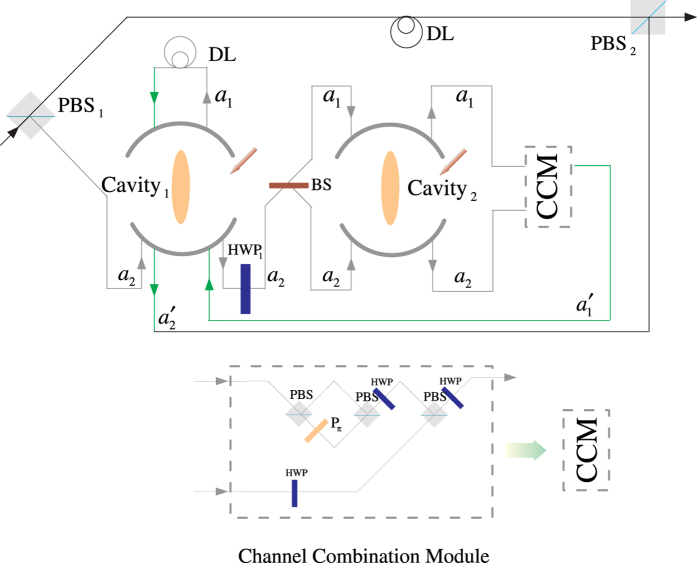
Scheme for implementing a three-qubit Toffoli gate with a flying photon polarization and a magnon qubit as the two control qubits and another magnon qubit as the target qubit.

The schematic diagram for implementing a deterministic three-qubit Toffoli gate is depicted in Fig. 3, which performs a NOT operation on the second atomic ensemble (the target qubit) if and only if the photon is in the state |*V*〉 and the first atomic ensemble is in the state 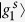. Suppose that the flying photon qubit is prepared in an arbitrary superposition state, 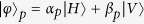, and each of the two independent atomic ensembles in cavities 1 and 2 is prepared in an arbitrary state as 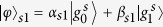 and 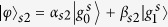. Here 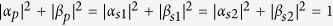.

First the photon reaches PBS_1_, the photon in the state |*V*〉 is injected into the cavity from path *a*_2_, while the photon in the state |*H*〉 does not interact with the atomic ensemble inside the cavity. With the same arguments as made for the CNOT gate above, we find that after the photon interacts with the atomic ensemble inside cavity 1, the state of the whole system evolves from 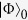 to 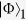. And


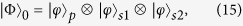



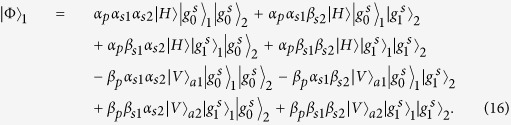


Then the photon from path *a*_1_ goes into a DL, while the photon from path *a*_2_ passes HWP_1_ and BS, and then gets into cavity 2 from path *a*_1_ or *a*_2_. Meanwhile we apply an H_*s*_ operation on the atomic ensemble in cavity 2. Considering the interaction between the photon and the atomic ensemble in cavity 2, we find the state of the system evolves from 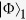 to 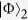, here


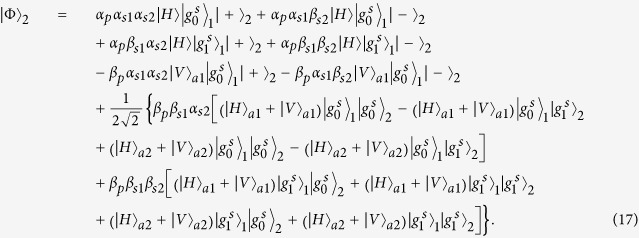


After the photon passes the channel combination module (CCM), we perform an H_*s*_ operation on the atomic ensemble in cavity 2 again, then the state of the combined system becomes


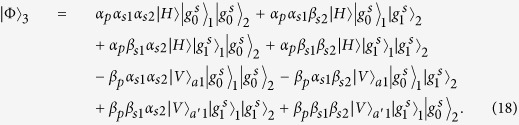


After the photon passes through the CCM, it is led back to cavity 1 from path 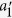, at the same time we lead the photon in path *a*_1_ into cavity 1 again (see the green lines), then the state of the system evolves into


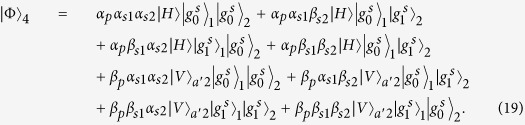


After the photon reaches PBS_2_, we can see that the state of the target magnon qubit in cavity 2 is flipped when the two control photonic qubit and the magnon qubit in cavity 1 are in the state |*V*〉 and 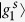, respectively. Therefore the quantum circuit shown in Fig. 3 can be used to construct a Toffoli gate on a photon-magnon hybrid system in a deterministic way.

### Fredkin gate on a three-qubit hybrid system

The three-qubit Fredkin gate implements a swap operation on two stationary atomic ensemble qubits in cavities 1 and 2 when the flying photon is in the state |*V*〉. Suppose that the initial states of the flying photon and the two atomic ensembles confined in the two double-sided cavities are


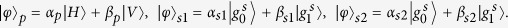


**Figure 4:**
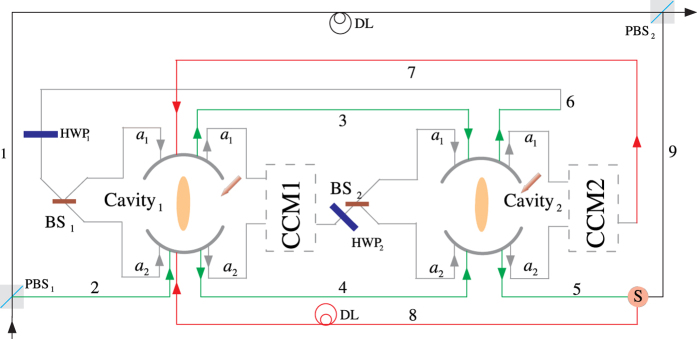
Schematic setup for a deterministic three qubit Fredkin gate with a flying photon polarization as the control qubit and two confined magnon qubits as the target qubits. S is an optical switch.

And 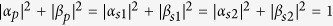. As illustrated in Fig. 4, our scheme for a three-qubit Fredkin gate can be achieved with three steps.

*Step 1*. The injected photon is split by PBS_1_ into two wave-packets, the photon in state |*H*〉 dose not interact with the atomic ensemble in cavity 1, while the photon in state |*V*〉 goes into path 2 and experiences the nonlinearities (see the green lines). After the photon in the state |*V*〉 is injected into cavity 1, the state of the three-qubit hybrid system changes to


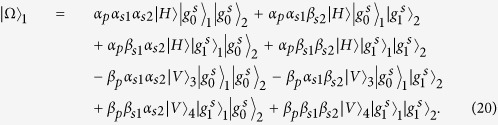


After the photon interacts with the atomic ensemble inside cavity 1, it emits from path 3 or 4 and then be led into cavity 2. After the photon interacts with the atomic ensemble inside cavity 2, 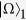 becomes


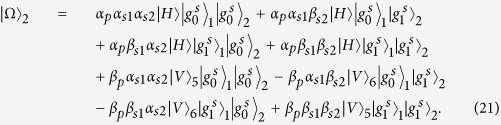


It can be seen that, when the photon in |*V*〉 passes through the two cavities in succession, the output path of the photon is determined by the parity of the two magnon qubits.

*Step 2*. The photon at S will be led to path 8, while the photon emitting from path 6 be led into cavity 1 again. As discussed above, in this round, the photon in path 6 acts as the control qubit and performs NOT operations on the magnon qubits in cavities 1 and 2, respectively (see the grey lines, i.e., HWP_1_ → BS_1_ → H_*s*1_ → Cavity1 → CCM1 → H_*s*1_ → HWP_2_ → BS_2_ → H_*s*2_ → Cavity2 → CCM2 → H_*s*2_). For this purpose, H_*s*_ operations on the atomic ensembles in cavities 1 and 2 before and after the photon interacts with the corresponding magnon qubit respectively are needed. When the photon emits from path 7, the output state of the system is


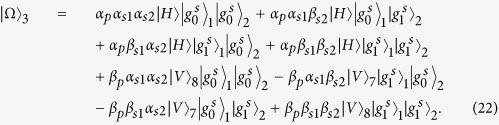


*Step 3*. In this round, the photon emitting from path 7 or 8 will be led into cavities 1 and 2 successively again. As discussed in *step 1*, after the photon interacts with cavity 2 again, the state of the system evolves into


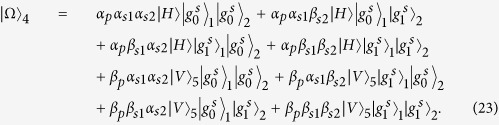


After this round, the photon emitting from path 5 will pass through S and reach PBS_2_. After the photon from path 1 or path 9 reaches PBS_2_, 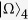 evolves into 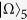,


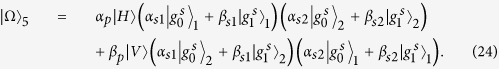


From Eq. (24), one can see that the states of the two solidstate target qubits (the two atomic ensembles in cavities 1 and 2) are swapped when the photon qubit is in the state |*V*〉, while they do not swap when the photon qubit is in the state |*H*〉. The quantum circuit shown in Fig. 4 can be used to construct the Fredkin gate on a three-qubit hybrid system in a deterministic way.

## Discussion

The key ingredient in our scheme is the combined magnon-cavity unit, such a system is a promising candidate for QIP since the birefringent propagation of the successively input photons acts as the effective Kerr nonlinearity. In this section, We quantitatively characterize the fidelities and efficiencies of our hybrid gates, respectively.

**Figure 5:**
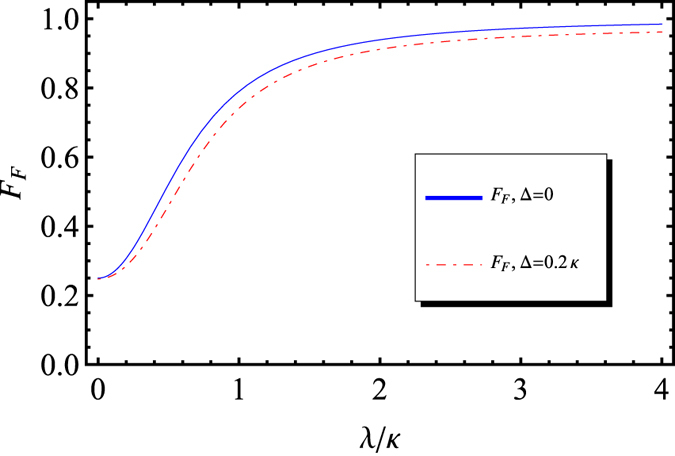
The fidelity of our Fredkin gate with symmetric double-sided cavities. The blue solid line stands for the resonant case and the red doted line represents the case with Δ = 0.2*κ*. Here *γ* = *κ* is taken for practical microcavity.

**Figure 6:**
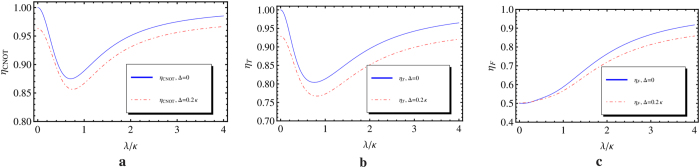
The efficiencies of our CNOT (a), Toffoli (b) and Fredkin (c) gates with symmetric double-sided cavities. The blue solid lines stand for the resonant case and the red doted lines represent the case with Δ = 0.2*κ*. Here *γ* = *κ* is taken for practical microcavity.

The fidelity of our Fredkin gate with respect to normalized photon detuning Δ/*κ* and the coupling rate *λ*/*κ* are shown in Fig. 5 when *γ* = *κ*. In principle, the detuning Δ/*κ* can be arbitrarily reduced, if the input photon is tuned to be resonant to the cavity, and then one has *F*_*F*_ = 97.2% when *γ* = *κ* and *λ*/*κ* = 3; while when photon detuning 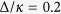 and 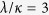, one has 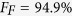. The fidelity *F*_*F*_ approaches a steady value limited by the frequency detuning 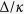. The efficiencies of our universal quantum gates are shown in Fig. 6 when setting *γ* = *κ*. For Δ = 0, *γ* = *κ* and 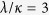, 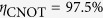, 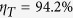, 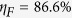; while when photon detuning 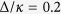 and 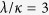, one has 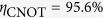, 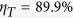, 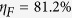. We can see that the performance of our universal quantum gates, to some extent, are not sensitive to the detuning Δ and get better when the coupling rate *λ*/*κ* increases.

**Figure 7:**
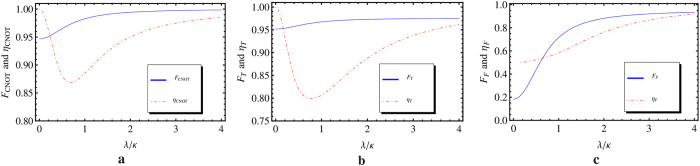
The fidelities and efficiencies of our CNOT (a), Toffoli (b) and the Fredkin (c) gates with asymmetric double-sided cavities. The blue solid lines stand for the fidelities, the red doted lines represent the efficiencies. Here the cavity coupling rate difference is chosen as *κ*_Δ_ = 0.1*κ*_*b*_, and we choose the detuning Δ = 0 and *γ* = *κ* for practical microcavity.

In fact, there might be some difference in the coupling rates between the cavity and modes 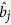 and 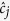 (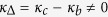) in practice. In experiment, the difference of the two coupling constants 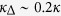 has been demonstrated, which yields approximately the same fidelity for both transmission and reflection directions^42^. In the resonant case (*ω*_*c*_ = *ω*_0_ = *ω*), there will be an additional error probability 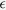 in the single-photon scattering process by 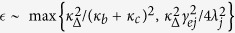. And this error can be improved for the cavity with almost identical mirrors^43^,^44^, which will lead to the ideal photon blockade^45^. To discuss the sensitivity of our schemes to *κ*_Δ_, the fidelities and efficiencies of our gates are calculated with the similar procedure as those used in the symmetric case by using the reflection and transmission coefficients obtained with the asymmetrical cavity. The fidelities and efficiencies of our gates are shown in Fig. 7, here we choose 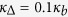, *γ* = *κ*_*b*_ and Δ = 0. When setting *λ*/*κ* = 3, one has 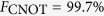 with 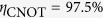, 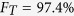 with 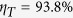 and 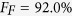 with 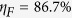. Compared with those in the symmetric case, the little decreases of the fidelities and efficiencies in the asymmetric case prove that our universal quantum gates are robust to the cavity coupling imbalance.

As reported in refs 46, 47, the maximum coupling strength between a single atom and a single intracavity photon, along with the decay rate of the excited state and the cavity mode, are 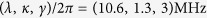. Thereby we can see that our hybrid quantum gates are robust against the practical imperfections. Recently, there have been plenty of other methods to couple an atomic ensemble with an optical cavity^48^,^49^, which might be another building block for our schemes. The fidelities of the spin wave rotation procedures of 99% have been reported^50^, and the collective spin wave operations in atomic ensembles have been well developed^51^. Besides, the atomic ensembles can store photons in a single atomic ensemble with several milliseconds^52^, so this manon-cavity unit is a good quantum memory system for photonic qubits, which is essential in scalable quantum networks. Therefore, our hybrid quantum gates may be achieved with the current QED setup. In addition, our hybrid quantum gates are quite different from the previous ones based on the quantum dot embedded in microcavities^6^,^7^ and those assisted by NV centers embedded in photonic crystal cavities coupled to two wave guides^30^. We use the atomic ensemble approach, so that light-matter coupling is largely improved by collective enhancement^53^. The control qubit of our gates is encoded on the polarization of the moving single photon and the target qubits are encoded on the magnon states of the atomic ensembles inside optical microcavities. As discussed in Sec. III, when the photon in |*V*〉 passes through the two cavities in succession, the output path of the photon is determined by the parity of the two magnon qubits, this makes the present schemes more succinct than the previous schemes^6^. In addition, because they do not require that the transmission for the uncoupled cavity is balanceable with the reflectance for the coupled cavity, our schemes are robust, this is different from the hybrid gates which are encoded on the atom confined in a single-sided cavity^18^,^31^.

## Conclusion

In conclusion, we have designed the compact quantum circuits for implementing deterministic universal hybrid quantum gates, including the CNOT, Toffoli, and Fredkin gates, by means of the the effective Kerr nonlinearity induced by an atomic ensemble embedded in a double-sided cavity. The spontaneous emission and the cavity decay induce the different transmittance or reflectance coefficients between the hot cavity and the cold cavity in a magnon-cavity system. We have shown the schemes are robust to the variation of coupling rate *λ*_*j*_ and the detuning Δ involved in the practical experiments. High fidelities and efficiencies can be achieved in the strong coupling regime in our schemes. We hope this work will be useful in quantum computation and quantum networks with single photons.

## Methods

Under the ideal case, suppose that the optical elements, such as PBS, HWP, *P*_*π*_, and optical switch, are perfect, both the success probability and the fidelity of the present schemes are 100% in principle. For a practical magnon-cavity unit, the spontaneous emission of the collective states and cavity decay may leading to photon loss, which will reduce the performance of our hybrid gates.

### The fidelities of the gates

We introduce the gate fidelity, which measures the distance for quantum information, is defined as^54^


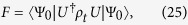


where 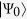 is the input states, *U* is the ideal CONT (Toffoli or Fredkin) gate, and 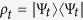, with 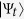 being the final state after the realistic CONT (Toffoli or Fredkin) operation in the present scheme. Considering the rules for optical transitions in a realistic cavity system, combing the arguments made in Sec. III, we find that the state of the system described by Eq. (12) becomes


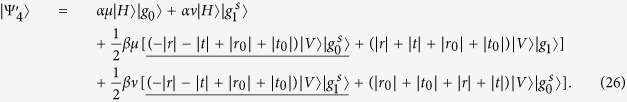


The terms with underlines indicate the states which take the bit-flip error. Then, the fidelity of the CNOT gate can be written as


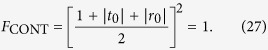


Similarly, we can calculate the fidelities for the Toffoli (*F*_*T*_) and the Fredkin (*F*_*F*_) gates discussed in Sec. III, respectively:


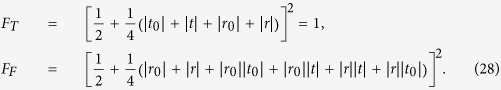


Defining the efficiency of a quantum gate as the ratio of the number of the outputting photons to the inputting photons. The reflection and transmission coefficients of the magnon-cavity system will modify the output states of the quantum gates. According to the discussions made in Sec. III, the efficiencies of our gates can be written as


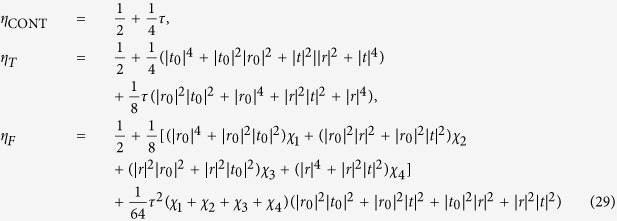


with  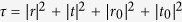, 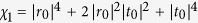, 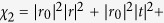 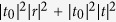, 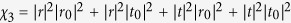 and 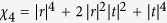.

### Experimental realization of an atomic ensemble cavity system

The physical configuration that we consider in the present schemes can employ ^87^Rb^55^,^56^ atomic ensemble. In a real experiment, one can couple a Bose-Einstein condensate of ^87^Rb atomic ensemble to an optical Fabry-Perot cavity^46^,^47^. We choose the two stable hyperfine ground states |*g*_0_〉 and |*g*_1_〉 as the (*F* = 1, *M*_*F*_ = −1) level and the (*F* = 1, *M*_*F*_ = 1) level of the 5*S*_1/2_ state, while two metastable hyperfine excited states are the (*F* = 2, *M*_*F*_ = −2) level and the (*F* = 2, *M*_*F*_ = 2) level of 5*P*_1/2_. Meanwhile, a highly excited Rydberg state *nS*_1/2_ can be chosen as |*r*〉.

## Additional Information

**How to cite this article:** Liu, A.-P. *et al*. Universal quantum gates for hybrid system assisted by atomic ensembles embedded in double-sided optical cavities. *Sci. Rep.* **7**, 43675; doi: 10.1038/srep43675 (2017).

**Publisher's note:** Springer Nature remains neutral with regard to jurisdictional claims in published maps and institutional affiliations.
